# Treatment of a recurrent pulmonary artery leiomyosarcoma with a combination of surgery, chemotherapy, and radiotherapy: A case report and literature review

**DOI:** 10.3892/ol.2015.2957

**Published:** 2015-02-11

**Authors:** YIN LV, FAN WANG, WENCHUAN QIAN, GUOPING SUN

**Affiliations:** Department of Radiotherapy, The First Affiliated Hospital of Anhui Medical University, Hefei, Anhui 230022, P.R. China

**Keywords:** pulmonary artery, leiomyosarcoma, radiotherapy, pulmonary thromboembolis

## Abstract

Pulmonary artery (PA) leiomyosarcoma (PAL) is a rare but extremely malignant tumor of the cardiovascular system, which is often misdiagnosed as pulmonary thromboembolism. Although the early detection and surgical resection improves patient survival, the benefits of adjuvant therapy are not well understood. The current study presents the case of a patient with primary PAL who underwent surgical resection along with three courses of chemotherapy. Despite these interventions, the patient experienced recurrence of the PAL within four months following treatment. The patient received localized radiotherapy and subsequently achieved a stable disease state. This case indicates that radiotherapy may offer a benefit to PAL patients, particularly those who do not respond to chemotherapy.

## Introduction

Pulmonary artery leiomyosarcoma (PAL) is an extremely rare primary sarcoma of the pulmonary artery with an incidence rate of ~0.001–0.03% (1,2). Due to its low incidence, PAL is often undetected and frequently misdiagnosed as chronic pulmonary embolism (3,4). However, the majority of PAL tumors are highly malignant, highlighting the requirement for improved methods of detecting and treating these tumors (2). Early detection and surgical resection of the primary PAL lesion have been demonstrated to improve patient survival; the median survival time of all patients was 1.5 months, but this was prolonged to 10 months with surgery such as pneumonectomy or mere excision of the tumor from the vascular bed (5). However recurrence of PAL often occurs following surgical resection (6). The benefits of adjuvant therapy for PAL tumors have not been clearly defined, particularly in the case of recurrent tumors. A previous report showed that a patient with primary leiomyosarcoma, who was receiving adjuvant therapy, survived for as long as 20 months (7). The current study presents the case of a patient with recurrent PAL. Written informed consent was obtained from the patient.

## Case report

A 42-year-old male presented to The First Affiliated Hospital of Anhui Medical University (Hefei, China) with repeated and progressive dizziness and chest pain for the previous five months, and with one occurrence of syncope in this period. The patient was admitted to the Department of Cardiac Surgery on 25th October 2012. Upon physical examination the patient’s blood pressure was 100/70 mmHg (normal range, 100–140/60–90 mmHg; 1 mmHg=0.133 kPa) and his heart rate was 78 beats/minute. No cyanosis was detectable when the patient was in a supine position. The patient’s jugular vein filling time was normal, and a slightly enlarged left heart border was observed. A grade III systolic murmur was identified between the second and third left intercostal space. Cardiac exam indicated that the patient’s pulmonary valve closing (P2) was normal, with no signs of hyperactivity. The liver was not accessible for examination. Blood tests revealed no abnormalities in the patient’s blood cell counts or blood chemistry. Echocardiography (ECG) indicated that the right atrium and right ventricle had significantly increased in size. ECG also revealed abnormal Q waves of and ST-T changes in the II, III and aVF leads. The pulmonary trunk and left distal pulmonary artery were filled by light reflection. The patient experienced severe tricuspid regurgitation, with estimated pulmonary artery pressure of 126 mmHg (normal range, 12–18 mmHg).

**Figure 1:**
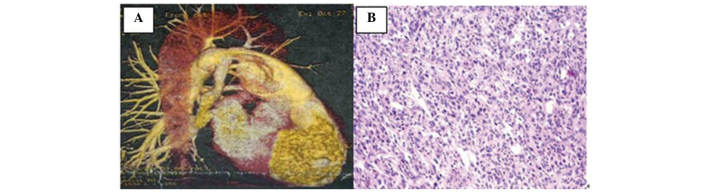
(A) Major vascular reconstruction was performed by Computed tomography angiography (10/27/2012). (B) Representative pathological image following surgery on 11/14/2012. Tissue was stained using hematoxylin and eosin.

**Figure 2:**
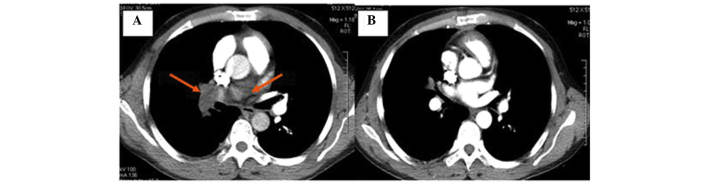
A representative computed tomography image (A) prior to surgery (10/27/2012), and (B) following surgery (11/30/2012). Arrows indicate the location of the pulmonary artery leiomyosarcoma tumor prior to surgery.

Based on these symptoms, an initial diagnosis of pulmonary thromboembolism with severe pulmonary hypertension and a minor pericardial effusion was determined. A computed tomography (CT) angiogram revealed thrombosis of the pulmonary artery, left pulmonary artery, and the right pulmonary artery branch. The diameters of ascending aortic and descending aortic were 28.9×30.9 mm and 23.5×23.4 mm, respectively (normal diameters, <35×35 mm) (Figs. 1A and 2A). A vascular ultrasound indicated that the portal vein, splenic vein, inferior vena cava, and bilateral iliac vein of the patient were all normal.

Based on the initial diagnosis of pulmonary thromboembolism, the patient was scheduled to undergo a pulmonary embolectomy in combination with tricuspid valvuloplasty under extracorporeal circulation. However, during surgery, a mass predominantly involving the right pulmonary artery and its branches was observed. No similar mass was identified in the left pulmonary artery. Tissue samples of the mass were extracted from the patient and frozen for subsequent analysis. Analysis of the frozen tissue section indicated that the mass was a malignant emboli of unknown origin. Therefore, tumor cystectomy and tricuspid valvuloplasty was performed.

Upon postoperative pathological analysis, the mass was identified as a spindle cell sarcoma, indicating that the mass was a PAL tumor (Fig. 1B). Immunohistochemical analysis showed that the cells of the mass were positive for CD117 and negative for cytokeratin (CK) 7, CK20, Villln, CD34, DOG-1, CK10 and CD10. Proliferation analysis by Ki-67 staining indicated that 40% of the cells were actively proliferating in the tumor. Following the removal of the tumor, the patient’s dizziness and chest pain were eliminated and no abnormal lesions were detected in the right pulmonary artery under enhanced CT examination on 30th November 2012 (Fig. 2B). Following surgery, the patient received three cycles of chemotherapy with 100 mg epirubicin and 20 mg cisplatin under a d1-5 regimen on 11th December 2012, 12th January 2013, and 19th February 2013, respectively.

**Figure 3:**
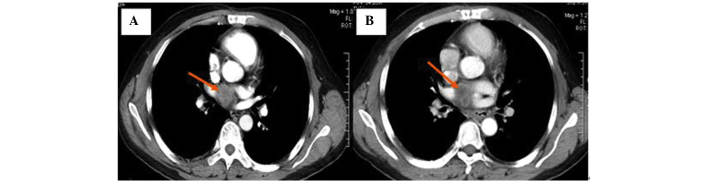
(A) Representative CT image (3/20/2013) showing recurrence of the pulmonary artery leiomyosarcoma tumor (arrow). (B) Representative CT image during radiotherapy (04/16/2013) showing a slight reduction in the size of the tumor (arrow). CT, computed tomography.

**Figure 4:**
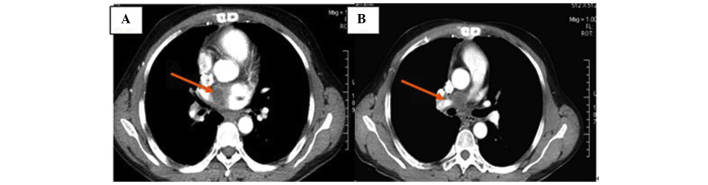
(A) Representative CT image after 25 days of radiotherapy at 5/27/2013. (B) Representative CT image recorded during follow up three months following radiotherapy (8/08/2013), illustrating further reduction of the tumor mass (arrow). CT, computed tomography.

**Figure 5:**
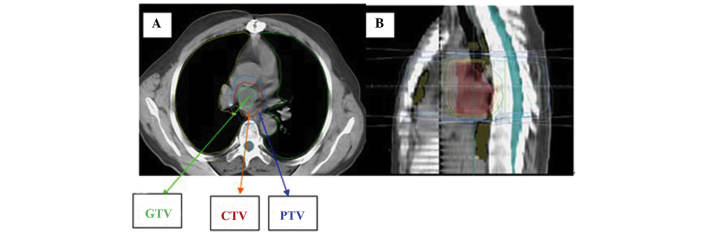
The planning of radiotherapy. (A) The target delineation and (B) the iso-dose curves of clinical target volume.

On 20th March 2013, the patient returned to the hospital presenting with dizziness and congestion. Enhanced chest CT examination revealed a soft tissue mass of 3.5×3.4×3.6 cm in size in the right pulmonary artery (Fig. 3A). The mass was diagnosed as a recurrent PAL tumor. The patient’s Karnofsky Performance Score (KPS) was 60, indicating that the quality of life was compromised (8). The patient was subsequently treated with intensity modulated conformal radiotherapy (diffusion-tensor; 32 Gy/16 f) at the right pulmonary artery between 28th March and 16th April 2013. Following therapy, the patient reported relief of dizziness and congestion. Enhanced chest CT examination revealed that the tumor mass had reduced in size to 3.1×3.2×3.3 cm (Fig. 3B). The patient received additional radiotherapy (30 sessions) with a dosage each time of 2 Gy administered five times a week for six weeks until May 2, 2013 (Fig. 5). During the course of radiotherapy, the patient reported chest pain when swallowing food, which was due to radiation esophagitis; this was relieved following symptomatic treatment (160,000 units gentamicin, three times a day, orally). The patient did not receive any additional chemotherapy after radiotherapy. By 27th May 2013, enhanced chest CT examination indicated that the mass had further reduced in size to 3.0×3.2×2.7 cm (Fig. 4A). Following this course of radiotherapy, the patient reported relatively good health with minor tightness of the chest after climbing stairs quickly, and had regained the ability to perform mild to moderate physical labor. Enhanced chest CT examination on 8th August 2013 showed further reduction of the PAL mass to a size of 2.5×2.2×2.4 cm (Fig. 4B). Bronchial ultrasound examination of the patient’s abdomen revealed no further abnormalities. The patient was followed up four times over a period of eight months. The tumor appeared to be stable for the first three sessions. However, at the fourth follow-up tumor growth was observed. At this time, the patient’s quality of life was substantially improved, with a KPS of 90.

## Discussion

Primary leiomyosarcoma of the pulmonary artery is an extremely rare tumor, and the majority of PAL patients present with non-specific symptoms including dyspnea, chest pain, coughing and hemoptysis (5,9). As a result, many PAL patients are initially misdiagnosed with pulmonary thromboembolism (10–13), as demonstrated in the present case. However, as PAL is an extremely aggressive tumor, more thorough diagnoses must be considered when patients present with such symptoms, so as to minimize the risk of misdiagnosing a malignant PAL lesion. In particular, the diagnosis of a tumor must be considered in the absence of predisposing factors for thromboembolism, in cases were the patient’s symptoms persist or recur despite adequate anticoagulation, and also in cases with a unilateral distribution of a massive perfusion defect (14).

In the current study, PAL was diagnosed incidentally during surgery and the tumor was subsequently resected, which potentially contributed to the favorable prognosis of this patient, as early detection and surgical resection has been shown to improve PAL survival rates (6). Patients typically develop PAL between the ages of 40 and 60 years. Thus patients within this age range should be subjected to a thorough clinical examination, and diagnoses including PAL, thromboembolism and lung cancer must be considered. For maximal benefit, definitive diagnostic methods must be employed to differentiate between these possibilities, and immediate surgical intervention must be performed in cases of cancer.

The diagnosis of PAL is frequently delayed due to the nonspecific symptoms, therefore, these neoplasms are often well developed before they are clinically diagnosed (2,9,15,16). If the tumor is detected early and can be completely resected, surgery offers the best opportunity to eliminate PAL without residual disease. However, despite aggressive surgical intervention, the mean patient survival time of 1.5 months has been shown to improve by only a few months, to 10 months, due to the aggressive nature of advanced PAL lesions (5,17). Another study showed that the median survival time of all patients was 1.5 months, but this was prolonged to 10 months following surgery such as pneumonectomy or mere excision of the tumor from the vascular bed (5). This is also shown in the current study, as the PAL recurred within four months of surgical resection, indicating that the patient’s disease had already reached an aggressive state at the time of diagnosis.

To date, little evidence is available indicating that adjuvant therapies, such as chemotherapy or radiotherapy, are beneficial for patients with recurrent PAL (6,18–20). Furthermore, only a few reports have demonstrated the successful treatment of recurrent PAL, primarily through surgery or stenting (6,19,20). Notably, in the current study, radiotherapy was found to effectively inhibit and control the development of PAL in the patient. This case suggests that radiotherapy may benefit PAL patients, including those who have failed to respond to surgery and/or chemotherapy. However, why radiotherapy was effective in this case, but ineffective in previous reports, remains unclear. A more extensive analysis of the factors that influenced the present patient’s response to radiotherapy may aid in informing future treatment strategies for recurrent PAL.

Chemotherapy and radiotherapy are two major adjuvant therapeutic methods for the treatment of malignancies (21,22). The therapeutic efficacy of these two methods varies depending on the specific tumor. In the present case, the patient did not respond well to chemotherapy, however, did respond to radiotherapy. This is in contrast to the majority of reports describing PAL, in which adjuvant therapies had been largely ineffective, as PAL is considered not sensitive to radiotherapy and chemotherapy. Therefore, there is insufficient evidence to conclude that radiotherapy is more efficient than chemotherapy in treating recurrent PAL. Nonetheless, the clinical results presented in this case report indicate that the use of radiotherapy to treat PAL warrants further investigation to better assess the efficacy of this therapeutic strategy.
